# The 3D Controllable Fabrication of Nanomaterials with FIB-SEM Synchronization Technology

**DOI:** 10.3390/nano13121839

**Published:** 2023-06-11

**Authors:** Lirong Zhao, Yimin Cui, Junyi Li, Yuxi Xie, Wenping Li, Junying Zhang

**Affiliations:** School of Physics, Beihang University, Beijing 100191, China; lrzhao@buaa.edu.cn (L.Z.); cuiym@buaa.edu.cn (Y.C.); 18377327@buaa.edu.cn (J.L.); xieyuxi0214@buaa.edu.cn (Y.X.)

**Keywords:** 3D controllable fabrication, FIB-SEM synchronization system, ion–sample interaction, high aspect ratio

## Abstract

Nanomaterials with unique structures and functions have been widely used in the fields of microelectronics, biology, medicine, and aerospace, etc. With advantages of high resolution and multi functions (e.g., milling, deposition, and implantation), focused ion beam (FIB) technology has been widely developed due to urgent demands for the 3D fabrication of nanomaterials in recent years. In this paper, FIB technology is illustrated in detail, including ion optical systems, operating modes, and combining equipment with other systems. Together with the in situ and real-time monitoring of scanning electron microscopy (SEM) imaging, a FIB-SEM synchronization system achieved 3D controllable fabrication from conductive to semiconductive and insulative nanomaterials. The controllable FIB-SEM processing of conductive nanomaterials with a high precision is studied, especially for the FIB-induced deposition (FIBID) 3D nano-patterning and nano-origami. As for semiconductive nanomaterials, the realization of high resolution and controllability is focused on nano-origami and 3D milling with a high aspect ratio. The parameters of FIB-SEM and its working modes are analyzed and optimized to achieve the high aspect ratio fabrication and 3D reconstruction of insulative nanomaterials. Furthermore, the current challenges and future outlooks are prospected for the 3D controllable processing of flexible insulative materials with high resolution.

## 1. Introduction

Generally, nanomaterials have a size ranging between 1 and 100 nanometers, or at least one dimension within the order of nanometer. As their characteristic size shrinks to the micro- and nano- scale, nanomaterials exhibit unique physical and chemical properties because of volume effects, surface interface effects, quantum size effects, and quantum tunneling effects. They show excellent performances in many fields due to their mechanical properties, special magnetic behavior, high electrical conductivity, and high catalytic reactivity [1,2], which make them popular in microelectronics, biology, chemical engineering, medicine, energy, aviation, and aerospace, etc. [3,4,5]. As the properties of nanomaterials depend on their 2D/3D structures, characteristic size, and surface morphology directly, producing high-quality micro–nano structures with a high throughput at low costs has become a hot issue in recent years [6,7].

Nanomaterials with specific structures and functions can be prepared through the methods of “bottom-up” and “top-down”. The “bottom-up” approach mainly includes self-assembly and dip-pen nanolithography (DPN), where atoms and molecules are used as basic units to assemble and build specific structures. Self-assembly [8] can process nanomaterials with high resolution and low cost, but it has poor control in terms of feature size and the synthesis procedure. DPN [9] can achieve micro–nano structures by transferring molecules from the atomic force microscope (AFM) tip to the substrate, but it is too slow for mass production and limited to a few specialized devices. On the other hand, the “top-down” approach involves essentially cutting the bulk material to achieve a progressively smaller structure with a higher controllability [10], which includes mask lithography, mask-less lithography, thin film technology, and nanoimprint lithography (NIL), etc. Mask lithography is a typical “top-down” method with high precision and controllability, which consists of optical exposure, ultraviolet (UV) lithography, X-ray lithography, and electron beam lithography (EBL). Mask-less lithography includes laser direct writing and focused ion beam (FIB) fabrication, etc. Thin film technology mainly involves physical vapor deposition (PVD), chemical vapor deposition (CVD), and electrochemical deposition (ECD). Normally, NIL [11] can be composed of the mask preparation and pattern transferring from the mask to the substrate. Among all the machining technologies of “top-down” approach, FIB has become popular for the high-precision fabrication of nanomaterials, with the advantages of having a high resolution and multi functions, as it can shorten the processing period and improve material utilization by avoiding a series of complex processes in mask lithography. Moreover, FIB is compatible with semiconductor techniques, which is helpful for the circuit integration, complexity, and miniaturization of microelectronic nanomaterials. In this paper, FIB-SEM synchronization technology is reviewed to show its capacity for realizing the 3D controllable high-resolution fabrication of nanomaterials from conductors to semiconductors and insulators.

This paper firstly describes FIB technology in detail, including ion optical systems, the three operating modes of FIB, and the combining equipment with other systems. Then, the progress of a FIB-SEM synchronization system to achieve 3D controllable fabrication of conductive, semiconductive, and insulative nanomaterials with a high precision is reviewed, especially in 3D FIB-induced deposition (FIBID) nano-patterning, nano-origami, 3D milling with a high aspect ratio and 3D reconstruction. Finally, the existing challenges and future trends are prospected for the 3D controllable fabrication of flexible insulative materials.

## 2. Introduction of FIB Technology

FIB was developed in the United States in the late 1970s with the invention of the liquid metal ion source (LMIS) [12]. From then on, FIB has developed by improving the optical systems, expanding its application, and integrating with other systems. In the late 1980s, the eutectic alloy ion source promoted the performance of FIB greatly and expanded its applications in microelectronics, material analysis, device characterization, and other fields [4,13,14,15]. In the 1990s [16], SEM was combined with FIB to reduce sample damage and obtain sample imaging with a higher resolution. In recent years, it has become an important tool in the 3D fabrication of nanomaterials, as it can realize in situ milling, deposition, and implantation with a high precision. The following will present the development of FIB in detail. 

### 2.1. FIB Optical System

#### 2.1.1. Ion Sources

**Figure 1:**
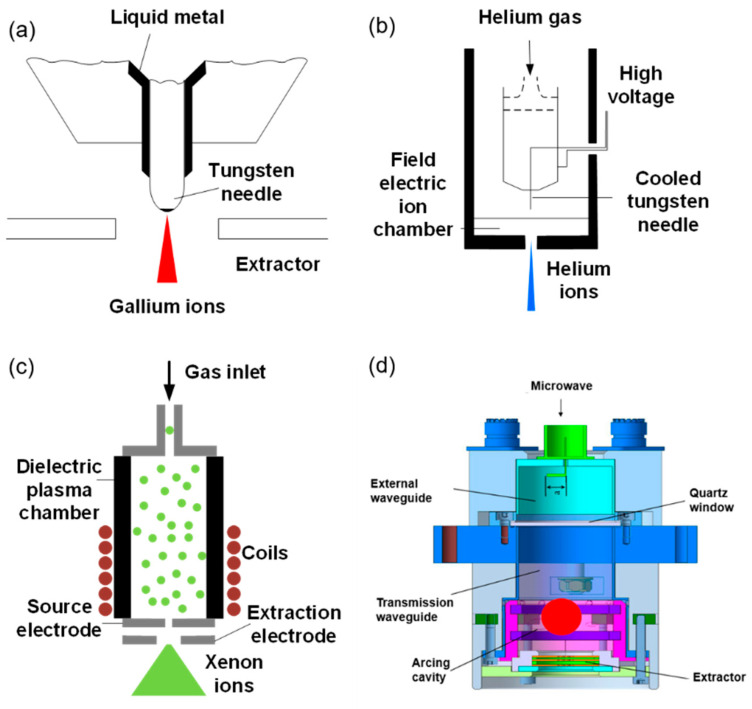
(**a**–**d**) Schematic diagrams of liquid metal ion source (LMIS) Ga, gas field ionization source (GFIS) He, inductively coupled plasma (ICP) source Xe, and electron cyclotron resonance (ECR) plasma ion source, respectively.

As the origin of the ion beam, the performances of ion sources determine the current and spot of FIB at a sample directly. There are many parameters for characterizing this ion source, such as the emission type, brightness ${\;\beta }_{r}$, energy spread $\Delta E_{\mathrm{FWHM}}$, source spot, and stability. Table 1 shows the performances of the current commercial ion sources. Among them, the LMIS gallium ion source is mostly used due to its low melting point (~30 °C), low volatility, low vapor pressure, high emission, and low surface free energy [17]. Compared with other ion sources, the Ga^+^ source has a relatively simple structure, as shown in Figure 1a. The tip is also called a Taylor tip, which is formed via ion emission from a liquid metal under the strong electric field. The Ga^+^ source can meet the requirements of submicron fabrication, as its source spot is about 50–100 nm, its brightness can reach $10^{6}\;{\mathrm{Am}}^{-2}{\mathrm{sr}}^{-1}V^{-1}$, and its emission is very stable. Generally, it can work at 3000 µA·h.

As for the gas field ionization source (GFIS) of lighter ion species, its brightness can be up to $10^{9}{\;\mathrm{Am}}^{-2}{\mathrm{sr}}^{-1}V^{-1}$ with a smallest source spot of 1 nm, but it needs an ultra-high vacuum and low temperature conditions. GFIS uses a strong electric field to ionize gas atoms or molecules (Figure 1b). Instead of providing a Ga ion with a liquid metal storage tank, GFIS originates from an inert gas supply system. Furthermore, GFIS can produce various ions, such as He^+^ and Ne^+^, which expands its potential applications [18]. 

Taking advantages of high ionization, high beam strength, low working pressure, and high stability, plasma sources of heavier ion species (e.g., Xe^+^ and Ar^+^) have been developed recently for large-volume/large-area milling. There are two ways to obtain plasma sources with a large emission current. One is inductively coupled plasma (ICP) and the other is electron cyclotron resonance (ECR). Figure 1c shows the schematic diagrams of an ICP source, where radio frequencies (RF) are applied to external coils to create an azimuth-induced magnetic field for accelerating the plasma electrons to ionize the stored gas. This mechanism can be employed in various plasma gases (e.g., Xe^+^, Ar^+^ and $O_{2}^{+}$) with a plasma density of $1\times 10^{13}{\;\mathrm{cm}}^{-3}$ (Xe^+^) and brightness of $1\times 10^{4}{\;\mathrm{Am}}^{-2}{\mathrm{sr}}^{-1}V^{-1}$ [19]. ECR plasma sources can be achieved by heating working gases with a 2.45 GHz microwave for ionization (Figure 1d). The plasma density can achieve the order of $10^{11}{\;\mathrm{cm}}^{-3}$, and the energy spread is ~5 eV [20].

#### 2.1.2. Ion Optical Column

**Figure 2:**
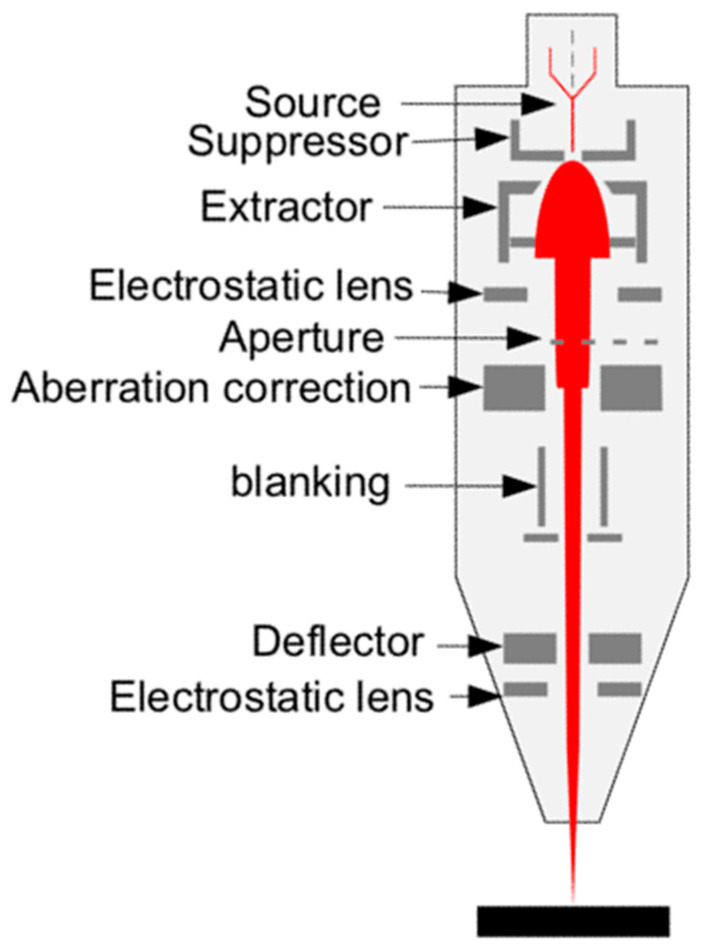
FIB ion optical column.

As shown in Figure 2, electrostatic units such as lenses and deflectors are chosen to accelerate, focus, and deflect the ion beam in a FIB micromachining system, as the mass of the ion is too large and magnetic units are not applicable. In the 1970s, the focusing system of a FIB was very single, with one lens primarily for ion imaging. It was developed to a two-stage lens system to obtain a higher resolution in the 1980s, which enlarged the FIB applications in micro-analyses, micro-machining, and material modification [15,21,22]. Compared to in-lens deflectors and post-lens deflectors, two pre-lens deflectors are mostly used to reduce the chromatic aberration, in combination with the dynamic focusing system and octupole stigmator [23]. For a FIB using the eutectic alloy ion source, an ion mass analyzer was developed to select the desired ions, such as Cobra E×B being used for a liquid metal alloy ion source (LAMIS) [24,25]. Since a gas injection system (GIS) was invented for a modern Ga LMIS FIB, the alloy ion source had become out of date.

Advances in the ion source and ion optical column have greatly improved the performance of FIB systems. For a modern commercial Ga LMIS FIB, the beam current ranges from several pA up to 100 nA and the beam diameter at the sample is less than 5 nm. The first commercial GFIS helium ion microscope (HIM) was launched by Zeiss in 2007, and it can realize an ultra-high processing resolution of 0.25 nm at the low current down to 0.1 pA [26,27]. Thermo Fisher SCIENTIFIC (Waltham, MA, USA) Xe (ICP)–FIB can now achieve a resolution of less than 20 nm (30 kV) at the coincidence for Helios 5 PFIB. Orsay physics released a Xe plasma source in 2011 and TESCAN (Brno, Czech Republic) launched the fourth generation FIB of Xe^+^ (ECR), achieving a resolution of 15 nm at 30 keV. Compared to Ga^+^ FIBs, He^+^ FIBs have better contrast [28] with less sample damage and a slower processing speed. Plasma source systems are well-suited for large-volume/large-area milling of up to hundreds of cubic microns [29,30], as their maximum probe current can reach 2 uA and they can process samples 50 times faster than Ga^+^ FIBs. 

### 2.2. FIB Operating Modes

**Figure 3:**
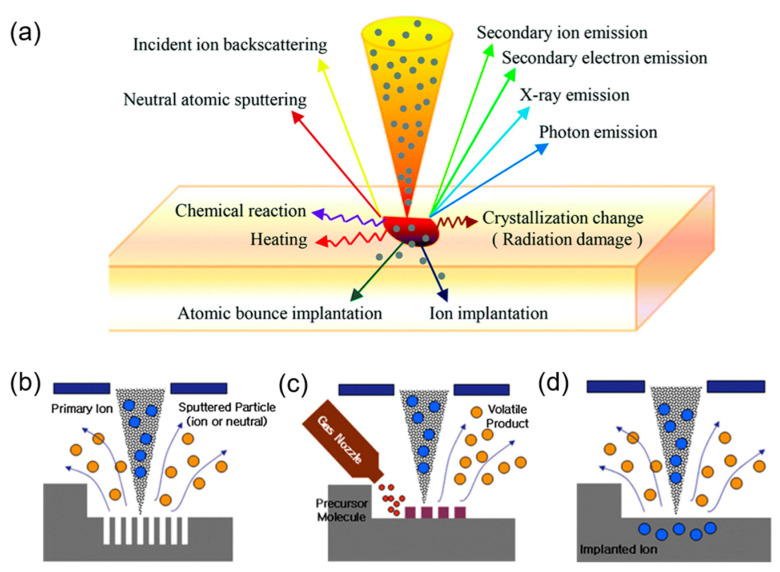
(**a**) Schematic of ion–solid interaction. Reprinted with permission from Ref. [32]. Copyright 2021, The Royal Society of Chemistry. (**b**–**d**) Basic operating modes of FIB processing of milling (**b**) milling, (**c**) deposition, and (**d**) ion implantation. Reprinted with permission from Ref. [33]. Copyright 2011, Elsevier Ltd.

The nature of a FIB’s multi functions (milling, deposition, and implantation) originates from ion–sample interaction, where the collision process involves a series of physical and chemical reactions illustrated in Figure 3a and the collision depth depends mainly on the ion energy. The main reactions include: (1) the sputtering of neutral atomic or molecules (fragments or clumps) in the form of positive or negative ions, (2) ion implantation or energy deposition, (3) a crystallization change (surface atoms gain momentum and move from their original position, leaving vacancies), (4) secondary ion sputtering, (5) secondary electron emission (for each incident ion, a secondary electron yield is about 10–1000 times more than a secondary ion one [31]), (6) heating (usually diffusing radially, longitudinally, or at multiple angles from the point of incidence), (7) photo emission, (8) incident ion backscattering, and (9) chemical reactions that involve the breaking of chemical bonds, thereby dissociating gas molecules (this effect can be utilized during film deposition). Based on the ion–solid interaction, a FIB can achieve milling, deposition, and ion implantation, as shown in Figure 3b–d. 

#### 2.2.1. Milling

Ion beam milling is one of the most widely used methods for preparing nanomaterials with specific nanostructures and properties. The five-axis stage can control the angle and height of the sample movement, allowing for the precise preparation of simple 2D graphics to complex 3D structures. FIB milling has been widely applied in inorganic materials, conductors, earth materials, optical fiber materials, biology materials, etc [3,34,35,36]. The initial application of a FIB includes a microwire analysis and the repair of the integrated circuit (IC) by connecting or cutting the wire on the semiconductor chip. FIBs play a great role in failure analyses and the repair of an IC by reducing the failure rate of 3 × 10^−9^. The global FIB industry is expected to grow from $1.3 billion in 2023 to $1.8 billion in 2028, with a growth rate of 7.0% annually. IC failure analyses will be the fastest growing market during this forecast period. In 2021, Thermo Fisher Scientific launched the Helios 5 EXL Wafer DualBeam in order to meet the increasing failure analyses performed in the semiconductor field. Recently, great progress has been made in FIB fabrication with atom probe tomography (APT) [37] and the transmission electron microscope (TEM) sample preparation of inorganic materials, metal alloys, crystals, and polymers, etc. [38,39]. By integrating APT, a sharp tip with a radius of curvature less than 50 nm can be formed using a sample thinning technique and the polishing of constantly small beams [37]. As the available TEM sample thickness using a FIB ranges from the micrometer scale to less than 50 nm [15], a 1–2 keV low-energy Ga ion beam or low-energy Ar ion beam [40,41] will be needed to decrease the sample damage induced by a high energy ion beam. GIS has been shown to enhance FIB etching by using auxiliary gases [26], such as Cl_2_, I_2_, and XeF_2_, to react with sputtering atoms and form gaseous substances. Different substrate materials can have varying enhancement coefficients [42], so it is very important to choose a suitable GIS. 

#### 2.2.2. Deposition

FIBID is an additive manufacturing technique for nanomaterials that enables the direct writing of the various nanostructures grown on the substrate surface with the assisted precursors. The above auxiliary gases in FIB milling are replaced by the organic precursor of a metal or semiconductor, such as C_2_H_5_Pt(CH_3_)_3_ and W(CO)_6_ [43], which can realize the FIB-induced micro/nano scale deposition of most metals or semiconductors. GIS-assisted shape modification includes nanowires (NWs), nanocolumn, nano-tweezers, and so on [44], which have been applied in various fields, including IC repair, optical lithographic mask repair, and grown specific functional structures [32,36]. FIBID is widely used in the detection and repair of ICs. For example, Lee et al. [45] successfully repaired damage by depositing Au as a microcircuit on the fracture of the IC with the precursor C_7_H_10_AuF_3_O_2_. Specific functional structures are usually grown in the form of 1D NWs or 3D nanocolumns, nanopillars, and nanohelices, which have special mechanical or electrical properties [46,47]. 

**Figure 4:**
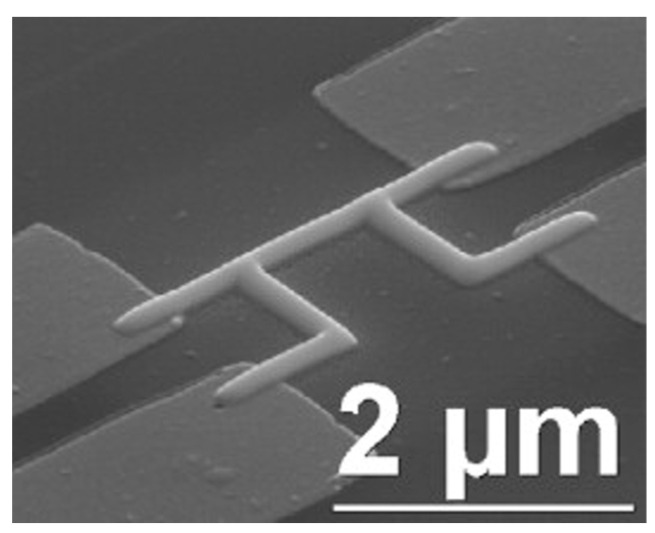
FIBID-based W nano bridge on SiO_2_ substrate. Reprinted with permission from Ref. [48]. Copyright 2007, Elsevier B.V.

Due to the comparatively large mass of a FIB, this inevitably results in sample amorphous ion implantation, and sputtering will happen with FIBID and side effects will appear. Normally, these side effects are useless. Now, researchers pay attention to find the novel electrical properties of these side effects that result from a combination of nanoparticles and impurities. For example, Luxmoore et al. [48] prepared FIBID-based W NWs on a SiO_2_ substrate (Figure 4a) and found that a single W NW superconducts at a temperature of 5.5 K. 

#### 2.2.3. Ion Implantation

**Figure 5:**
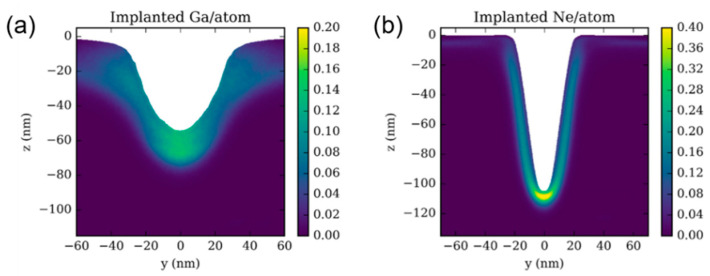
**D**istributions of **i**mplanted (**a**) Ga^+^ and (**b**) Ne^+^ in SiO_2_ substrate. Reprinted with permission from Ref. [54]. Copyright 2018, IOP Publishing Ltd.

Ion implantation is capable of changing surface compositions and structures to improve nanomaterials’ properties. The implantation depth can reach an average of 10 nm to 10 μm [49]. As shown in Figure 5, incident Ga^+^ and Ne^+^ with the same energy have different distributions in a SiO_2_ substrate. Ne^+^ ions are more concentrated in the range of about 10 nm below the surface, while Ga^+^ ions extend to a larger depth of approximately 20 nm due to their heavier mass. Ion implantation can be used in Raman enhancement, electrical conductivity, and the preparation of nanoscale quantum dots [47,50]. Because of these unique outstanding advantages, ion implantation has been widely used in semiconductor doping [51,52] and the surface modification of metals, ceramics, and polymers, etc. [53]. 

Ion implantation plays an important role in the preparation of opto-electronic devices by achieving good semiconductor doping with a sub-micron spatial precision. Deshpande et al. [55] investigated FIB-induced gallium doping on shallow silicon p-n junction devices in the micron-scale region and studied radiation damage, surface amorphous layer, and photoelectric performance. They concluded that formation of amorphous silicon layers can reduce the photoelectric performance of the diode. Phan et al. [52] obtained locally amorphous p-type silicon NWs that were thermally annealed at 700 °C through ion implantation and wet etching, and discussed the piezoresistive effect, which would accelerate the development of nano-electromechanical system (NEMS) sensors. Garg et al. [56] prepared 3D suspended micro–nanostructures of Si with a high aspect ratio (≈625) and small diameter (~31 nm), and they found that ion implantation caused the most amorphous properties in the prepared nanostructures.

Ion implantation has also been widely applied in the surface modification of nanomaterials. McKenzie et al. [57] discovered a 35 nm amorphous carbon layer and that the critical dose for amorphization of the diamond surface is 2 × 10^14^ Ga^+^/cm^2^. With higher doses, the amorphous layer can swell to 31% of its original volume and hold large amounts of gallium. Rubanov et al. [58] utilized a high-pressure, high-temperature (HPHT) annealing technique to graphitize the implantation layer in diamond by 30 keV Ga^+^ FIB. Wei et al. [59] prepared ordered 25–70 nm Ga nano quantum dots on a GaAs substrate without supplied gas. Furthermore, ion implantation can be also used for AFM tip preparation. Hu et al. [60] reported the first nanosphere probe manufactured by He^+^ implantation for accurately measuring the interface ranging from nanometers to microns, which would solve the technical bottleneck in nano-tribology.

### 2.3. FIB Equipment with Other Systems

As the mass of an ion is very large, some problems appear, such as a lower imaging resolution than SEM and sample damage, especially in crystal surfaces, existing in a single FIB system. In order to solve the above problems, other systems were induced to form dual-beam and multi-beam equipment. 

#### 2.3.1. FIB-SEM Dual-Beam System

**Figure 6:**
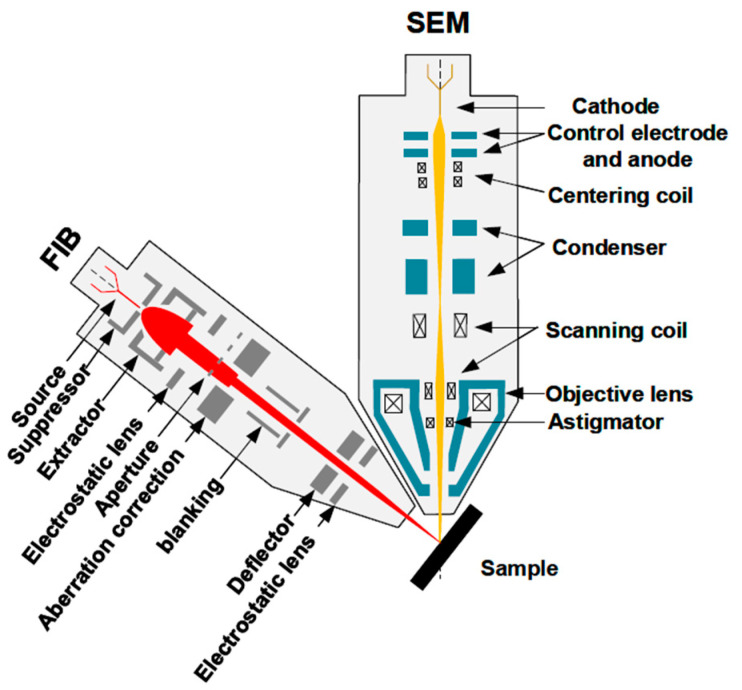
FIB-SEM dual-beam system.

Together with the in situ and real-time monitoring of SEM images, a FIB-SEM synchronization system maximizes the advantages of both ion and electron beams. As for the current SEM with Schottky Field Gun, it can last for 2000 h. Normally, a FIB-SEM synchronization system in a lab can work for more than 2 years without changing the ion source or the cathode. Two optical cylinders are typically connected at an angle of 50–60°, as shown in Figure 6. SEM can not only in situ monitor, but also neutralize ions to reduce the charge accumulation during FIB processing [61]. Moreover, 3D reconstructions can be performed by combing FIB repeatedly cutting cross-sections with SEM real-time imaging and the image processing software. Recent advances in FIB-SEM have enabled 3D reconstruction at a glancing angle, reducing image deformity due to tilt and providing more accurate representations of material structures. It has been successfully applied to the volume distribution of carbide in steel, crack tip analyses, the orthogonal cross-section characterization of tensile materials, carbon-based materials, bone hierarchical structures, proton exchange membrane (PEM) fuel cells, and so on [35,62]. Plasma FIB-SEM has emerged as a promising method for large-area milling, high-flux preparation with a low implantation layer, and high-throughput cryo-electron tomography. The use of plasma sources (e.g., Ar^+^ and Xe^+^) can achieve the 3D reconstruction of a larger area and volume of biomaterials [63,64]. Additionally, FIB-SEM is widely used in the characterization of material composition and 3D microanalyses, by combining with a mass spectrometer [65,66]. 

The traditional preparation methods for bulk TEM samples are mechanical thinning, electrolytic polishing, and the ion milling technique [15], and their low success rate limits the application of TEM. FIB technology effectively improves the success rate of TEM sampling up to 95% by combining real-time SEM monitoring and a nanomanipulator [67]. Combining with thinning technology, the quality of TEM sample preparation can be further improved by choosing a lower FIB energy, a low-voltage and low-angle broad ion beam, and plasma FIB-SEM [68]. Additionally, an optional TEM column can be mounted to realize in situ TEM observation. An improved STEM/TEM alloy sample can be achieved using Xe^+^ plasma FIB milling with less damage during ion implantation [69]. For ceramic materials with a high strength, high melting point, and poor toughness, ion beam thinning may cause damage such as cracks or holes, which will be exacerbated with heat accumulation on the surface. Therefore, it is necessary to thin the sample at a low rate with a low incidence angle and the appropriate ion energy (varying from material to material) [70]. 

In situ performance measurements can also be achieved by combining FIB-SEM with other attachments to fully understand and master the properties of nanomaterials. Micro heating plate devices can provide a stable and controllable environment for investigating materials at different temperatures, allowing for in situ observations of the thermal behavior, phase transformations, and crystallography and composition changes [71]. High density and uniformity in material preparations such as metals, ceramics, and composite materials can be obtained using in situ spark plasma sintering (SPS) technology with uniform heating and rapid cooling. The mechanisms of phase evolution during the sintering process can be achieved by integrating in situ SPS technology [72]. Combined with electron beam (EM) technology, the range of FIB applications has been greatly expanded for micro–nano electromechanical systems (MEMS/NEMS), site-specific device production, IC repairment, and complex TEM sample preparation [3,15,39,73]. In early FIB-SEM systems, FIB and SEM worked by switching, as the magnetic field of the SEM objective lens would affect the performance of the FIB greatly. A FIB-SEM synchronization system was developed by solving the magnetic leakage and it could realize the in situ and real-time high-resolution monitoring of FIB fabrication.

#### 2.3.2. Multi-Beam System

**Figure 7:**
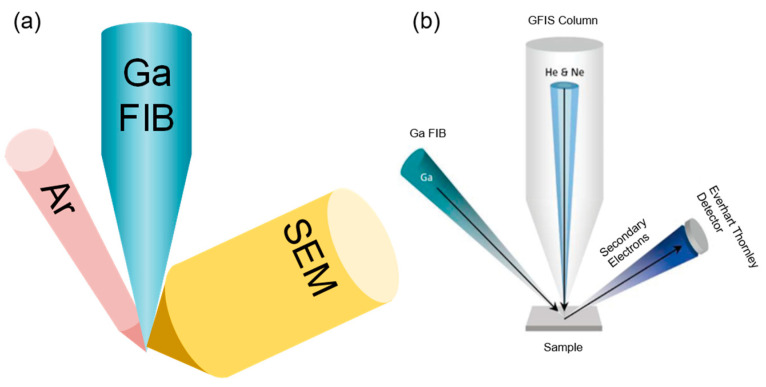
(**a**) Triple-beam system of FIB-SEM-Ar. (**b**) Ga-He-Ne multi-beam system. Reprinted with permission from Ref. [75]. Copyright 2013, AIP Publishing LLC.

Based on the FIB-SEM dual-beam system, a multi-beam system has been developed to reduce sample damage. The triple-beam system of FIB-SEM-Ar can switch in different multiple ion sources, as shown in Figure 7a. Hitachi (Tokyo, Japan) released the NX9000 with a selective add-on Ar ion column on FIB(Ga)-SEM in 2012, which effectively reduces this sample damage, as the damage thickness is only 2 nm for Ar^+^ instead of 10–30 nm for Ga^+^. Triple-beam technology realizes a continuous operation from high-precision FIB processing to Ar ion beam “cleaning” on one instrument, and greatly improves the sample preparation accuracy and reliability [74].

To further enhance the processing capacity of FIBs, Carl Zeiss implemented a multi-beam ion microscope named ORION NanoFab based on HIM, which integrated Ga, Ne, and He ion sources, as shown in Figure 7b. He and Ne share the same ion optical column to achieve fast switching. The nominal probe sizes of Ga^+^, Ne^+^, and He^+^ are 3.0, 1.9, and 0.5 nm, respectively [76]. ORION NanoFab has been successfully applied in magnetic materials, biology materials, and insulative materials, which has obtained graphene with direct-write features as small as 5 nm [77], surface plasma antennas with a tip gap of 4 nm [78], and line patterns with the line edge of 1.81 ± 0.06 nm and the width roughness of 2.90 ± 0.06 nm on Ni-based metal−organic clusters [79]. 

Compared to multi-beam systems, FIB-SEM dual-beam systems are more affordable. With advantages of a high precision, multi functions, and in situ real-time monitoring, FIB-SEM synchronization systems are expected to achieve the 3D controllable fabrication of nanomaterials.

## 3. 3D Controllable Fabrication of Nanomaterials with FIB-SEM

Based on the ion–solid interaction, the processing properties can be affected by the parameters of the incident beam and materials such as the ion energy, ion type, beam current, ion dose, dwell time, scanning strategy, material type, and composition. Simulating and optimizing these parameters with a computer is necessary for achieving a faster and more accurate fabrication of nanomaterials. The Monte Carlo (MC) simulation method is effective for evaluating these processing properties [33,80], which is based on the statistical law. At present, most MC simulations in FIBs can be realized using commercial software such as the Stopping and Range of Ions in Matter (SRIM) proposed by Ziegler et al. [81], TRINDY and TRI3DYN for a 1D layered dynamic model and 3D path simulations [82,83], SDTrimSP with 2D and 3D surface morphologies being considered [84], and EnvizION with FIB milling damage, secondary electron emission, and gas-assisted etching for Ga^+^, He^+^, Ne^+^ in Cu, W, and SiO_2_ [54,85,86,87,88,89]. Recently, artificial intelligence (AI) algorithms have provided guidance for selecting the parameters in pre-processing complex patterns with a >96% accuracy [90]. 

Now, FIB application ranges have expanded from conductors (>10^5^ s/m) to semiconductors (10^−7^–10^5^ s/m) and insulators (<10^−7^ s/m). When high-energy charged particles bombard samples, the sample conductivity may cause a local charge accumulation and affect the final fabrication, especially for insulative nanomaterials. The 3D controllable fabrication of conductive, semiconductive, and insulative nanomaterials using a FIB-SEM synchronization system is given in the following section. 

### 3.1. Conductive Materials

Most conductive materials have a high conductivity above 10^5^ s/m; thus, local sample charging can be neglected. Under the in situ real-time monitoring of high-resolution SEM, FIB parameters such as the ion species, energy, beam current, dwell time, and scanning strategy can be adjusted to achieve the 3D high-precision controllable fabrication of conductive nanomaterials. Their typical applications are described, including FIBID 3D nano-patterning (nanoelectrode, nanogap electrodes, and specific structures) and FIB milling (nano-origami). 

**Figure 8:**
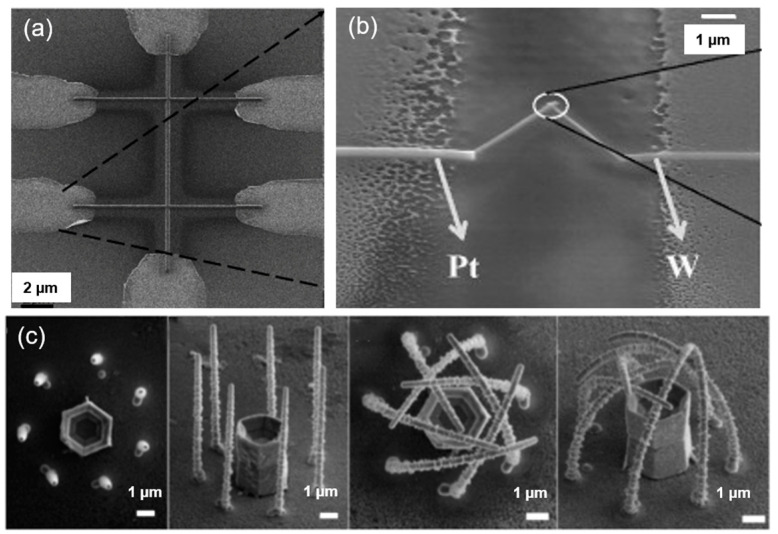
(**a**) Deposited devices prepared for temperature-dependent electrical conductivity measurements (scale bar: 2 μm). Reprinted with permission from Ref. [91]. Copyright 2013, IOP Publishing Ltd. (**b**) FIBID-based Pt-W nanothermocouple is used to measure the temperature near 100 nm on the substrate. Reprinted with permission from Ref. [92]. Copyright 2009, Elsevier B.V. (**c**) Nanocage construction with Pt wire for single crystalline ZnO tube immobilization (scale bar: 1 μm). Reprinted with permission from Ref. [93]. Copyright 2013, Spring Nature.

Nanoelectrodes can be prepared using FIBID-based deposited metal, which plays an important role in the measurements of electrical properties. Wu et al. [91] fabricated nanodevices with He^+^ FIB and Ne^+^ FIB for temperature-dependent electrical conductivity measurements, and they found that a different resistivity could be obtained by choosing a different FIB incident beam (He^+^ and Ne^+^), as shown in Figure 8a. Shukla et al. [92] succeeded in obtaining a faster-response Pt-W nanothermocouple by optimizing the FIB parameters to monitor the local temperature increase in a SiO_2_ substrate (Figure 8b). The nanothermocouple would react when the local temperature rose by 50 °C under 0.3 mW (30 kV, 10 pA) FIB irradiation. In Figure 8c, Cui et al. [93] grew Pt NWs on a freestanding functional entity using thermal annealing, then fabricated the nanocages of these Pt NWs by controlling their bending angle, with the growth parameters, annealing temperature, and annealing duration/cycle being adjusted. These nanocages could confine ZnO tubs, which show a great potential in the nanofabrication of large-area 3D functional devices for micro/nano object fixation. 

**Figure 9:**
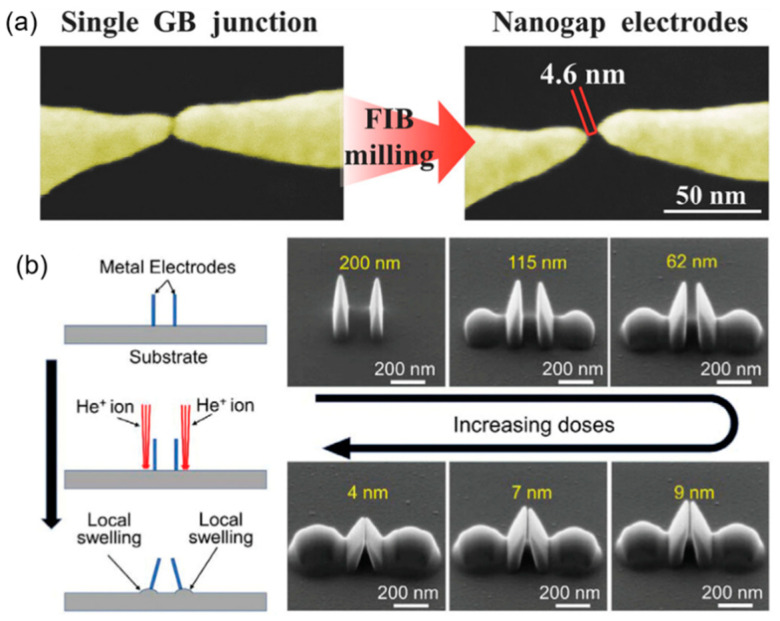
(**a**) SEM images of a single grain boundary (GB) junction before and after FIB milling. Reprinted with permission from Ref. [94]. Copyright 2015, Wiley-VCH. (**b**) Nanogap fine-tuning using substrate swelling induced by helium ion implantation. Reprinted with permission from Ref. [4]. Copyright 2022, Wiley-VCH.

Nanogap electrodes are an essential component in molecular device assembly, which are widely used in the fields of nanophotonics and nanoelectronics [32,47]. The width of nanogap electrodes can affect the amplified signals of sensors and demands for a higher controllability in fabrication. For example, Cui et al. [94] prepared suspended gold wires using electron beam mask lithography and then used FIB milling to create Au nanogap electrodes, as shown in Figure 9a. A small gap width of 4.6 nm was achieved by adjusting the FIB-SEM parameters. In Figure 9b, Wen et al. [4] deposited Pt electrodes and tuned the nanogap width using substrate swelling induced by He^+^ implantation. They were able to regulate the nanogap width to as small as 4 nm by increasing the ion doses.

**Figure 10:**
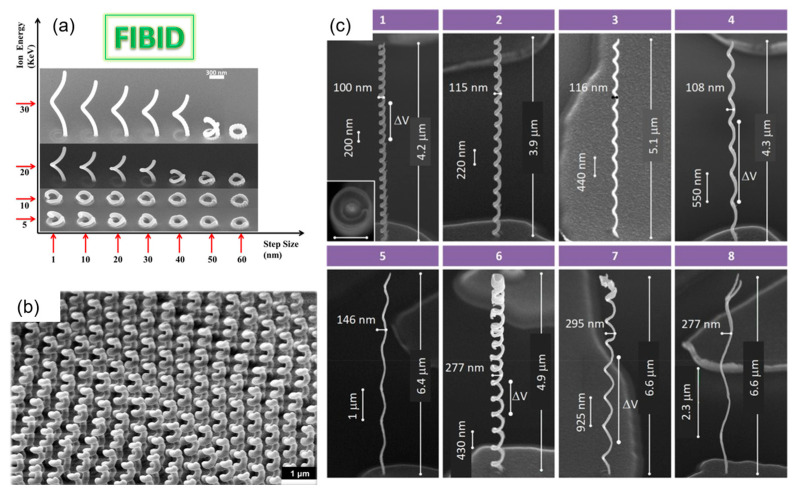
(**a**) SEM images of nanohelices under different beam energies and step sizes; and (**b**) array of 20 × 20 nanohelices fabricated by FIBID. Reprinted with permission from Ref. [97]. Copyright 2014, American Chemical Society. (**c**) W-C nanohelices grown by He^+^ FIB. Nanohelices of types 1–5 were grown by keeping the fixed nominal circular diameter of 75 nm and varying the beam dwell time from 700 to 2400 ms, while types 6–8 were grown by keeping the fixed nominal circular diameter of 200 nm and varying the beam dwell time from 650 to 2000 ms. Reprinted with permission from Ref. [98]. Copyright 2019, American Chemical Society.

Controllable FIBID 3D nano-patterning plays an important role in the preparation of specific structures and functional devices for optical and electrical applications. Wagner et al. [95] grew FIBID-based 3D multipod nanostructures by regulating the substrate temperature with a homemade Peltier stage. They found that FEBID at a low substrate temperature was not only faster, but also suited for high-fidelity 3D printing. Esposito’s team [96] prepared and studied the proximity and charge surface of chiral metallic nanospirals. This method has a high flexibility for the controllability of the geometry, array density, and size. Additionally, they also prepared 3D chiral plasmonic helices structures (see Figure 10a,b) by depositing Pt on a GaN/AlGaN substrate under different beam energies and step sizes [97]. They observed a highly selective dichroic band shifted toward shorter wavelengths with a maximum dissymmetry factor of up to 26% in the visible range. Cordoba et al. [98] deposited several types of W-C nanohelices by varying the diameter and beam dwell time of He^+^ FIB in 3D nanostructures (Figure 10c) with a fine superconductivity, high critical magnetic field, and current density. The nanohelices had dimensions of 100 nm in diameter and a high aspect ratio of up to 65.

**Figure 11:**
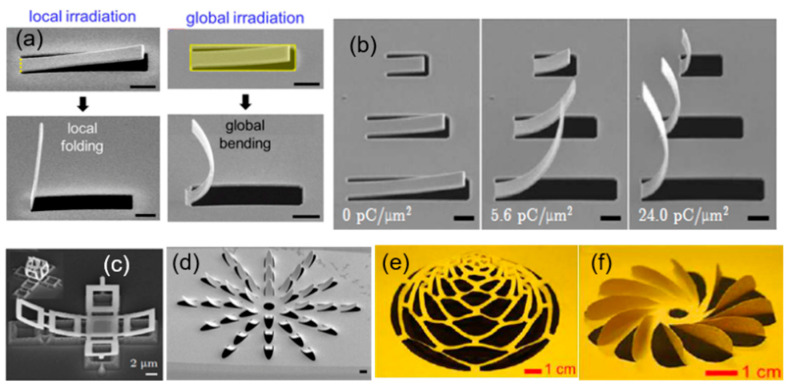
(**a**) The nano deformation is affected by irradiation mode. (**b**) The nano deformation is affected by ion dose. (**c**–**f**) Different 3D folding structures (**c**) metallic structures made of Al/Cr thin film before FIB irradiation. Inset: the final 3D structures after FIB irradiation; (**d**) a flower-shaped structure under global FIB irradiation; (**e**) origami of an expandable dome (corresponding to a traditional Chinese origami named “pulling flower”); and (**f**) a 12-blade propeller. (**a**,**d**) Reprinted with permission from [105]. Copyright 2018, AIP Publishing. (**b**,**e**,**f**) Reprinted with permission from [101]. Copyright 2018, American Association for the Advancement of Science. (**c**) Reprinted with permission from [106], Copyright 2011, Elsevier B.V.

Recently, a new technique for deforming nanomaterials called nano-origami was developed by combining FIB milling and ion beam irradiation [99]. This one-step, high-precision, micro–nano on-chip machining technique involves cutting and folding. Cutting is achieved through ion beam milling patterns and folding is realized by bending and twisting with ion beam irradiation, either locally or globally. Nano deformation (folding) is affected by the acceleration voltage, irradiation dose, and topological appearance of the material itself (Figure 11a,b). By adjusting these parameters, researchers have successfully prepared controllable 3D micro/nano functional structures with unique optical properties, such as an ultra-optical chirality, surface diffraction, phase and polarization regulation, and a photon spin Hall effect, as shown in Figure 11c–f [100,101,102]. This method shows potential for micro/nano photonic devices, microelectronics, MEMS, biomedicine, and other fields. For example, Liu et al. [100] prepared asymmetric 3D plasmonic structures with asymmetric split-ring resonators (SRRs) on a gold film (80 nm thick), which showed three Fano resonances. Nano-origami is suited for materials with a good conductivity, such as metal materials (e.g., Au, Al, and Ag) and semiconductors (e.g., Si, Si_3_N_4_, and graphene) [103,104]. 

### 3.2. Semiconductive Materials

Semiconductive materials usually have a conductivity of 10^−7^–10^5^ s/m, and an incident high-energy density FIB may cause local charging at their surface. Local sample charging can be greatly reduced by coating a conductive layer of Au/C, being transferred by the manipulator, and being neutralized with an electron beam [61]. Here, we will focus on the 3D controllable fabrication of semiconductive materials using FIB-induced deposition and FIB milling in nano-origami and high-aspect-ratio structures.

FIB-induced deposition plays an irreplaceable role in repairing optical and electronic devices. In general, different semiconductor materials of SiO_2_ and GaN can be deposited by choosing different precursors. Precursors such as tetramethylcyclotetrasiloxane (TMCTS), octamethyltetracyclosiloxane (OMCTS), pentamethylcyclopentasiloxane (PMCPS), dodecamethylpentasiloxane (DMPS), and tetraethoxysilane (TEOS) can be used for SiO_2_ deposition, while GaH_3_:NC_7_H_14_ can be used for GaN deposition [33]. Through controlling the gas density and FIB working parameters, Okada et al. [107] achieved a TEOS-induced deposition of SiOx at the 30 nm level to repair sag damage in UV mask plates. 

**Figure 12:**
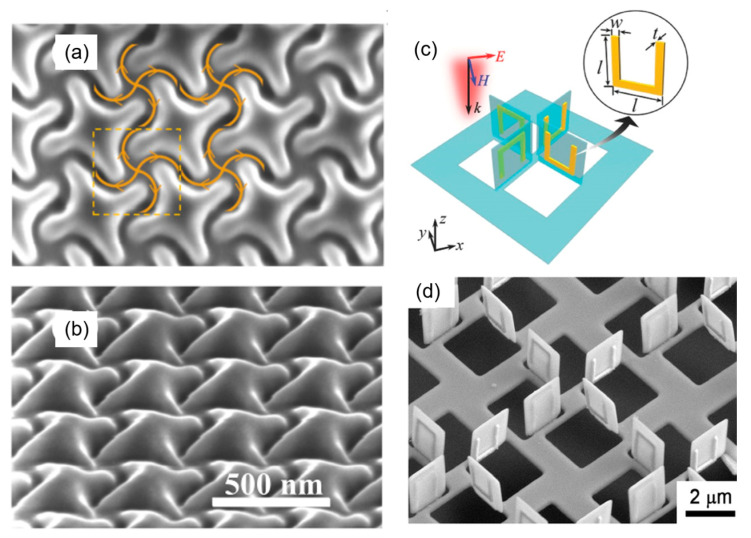
(**a**,**b**) SEM images of chiral patterning on Si/Al_2_O_3_ produced by digitally controlled FIB: (**a**) normal view with the FIB paths and directions (curved arrows) and the square unit cell (dashed boundary); and (**b**) SEM-image of the sample tilted by 52°; Reprinted with permission from [108]. Copyright 2018, Spring Nature. (**c**) Schematic diagram of toroidal molecule folded by ion beam. (**d**) SEM images of toroidal metamaterial array for SiNx. Reprinted with permission from [110]. Copyright 2017, WILEY-VCH Verlag GmbH & Co. kGaA.

For FIB milling, the 3D controllable preparation of nanostructures and devices with specific functions greatly promotes their applications in MEMS/NEMS, nano-optics, micro-energy, and other fields. Gorkunov et al. [108] prepared 3D structures on a 300 nm thin film of a monocrystalline epitaxial silicon on sapphire (Figure 12a,b) with a chiral nanoscale relief, by controlling the FIB scanning trajectory with the digital templates. The chiral metasurface structures showed a high transmittance (50–80%) with a circular dichroism of up to 0.5 and an optical activity of up to 20° in the visible range. Drezner et al. [109] fabricated the same structures on a (001) Si single crystal under a series of doses and measured the radius of the amorphization caused by a 30 kV Ga^+^ FIB. They found that the beam tail would influence the amorphization region for doses above 10^18^ ions/cm^2^. The amorphous damage caused by the beam tail could be reduced by using an appropriate dosage, thus achieving high-quality controllable fabrication. More applications of 3D controllable milling are shown in Table 2. Additionally, nano-origami can be achieved by combining FIB milling with ion irradiation. Liu et al. [110] prepared a 3D toroidal metamaterial with a SiNx sheet, whose side length was 1.7 μm and thickness was 100 nm (Figure 12c,d). High-quality factor toroidal resonance can be achieved using FIB-SEM synchronization technology, which shows its flexibility and nanoscale controllability for structure size, position, and direction, especially for 3D metamaterials.

**Figure 13:**
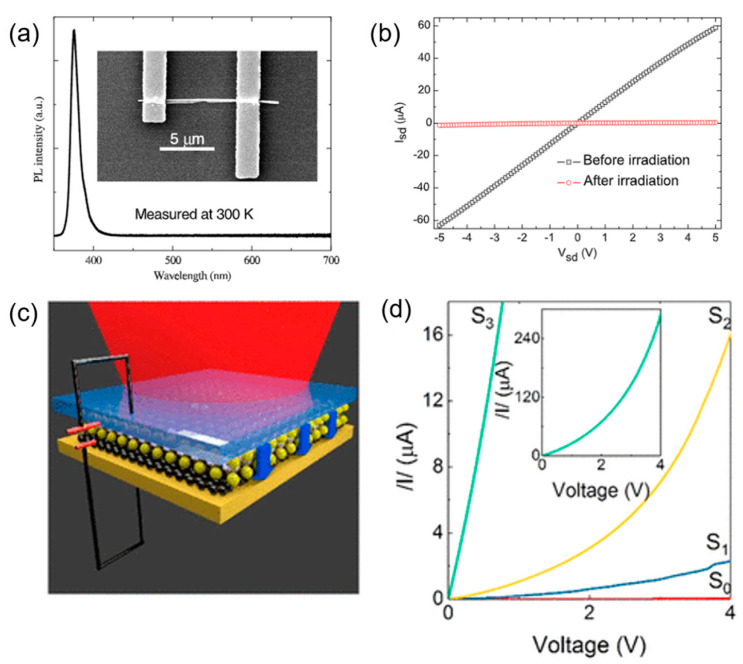
(**a**) The PL spectrum of single ZnO NWs measured at 300 K. Inset: an SEM image of the ZnO NW FET. (**b**) I_sd_–V_sd_ curves before and after ion irradiation. Reprinted with permission from [117]. Copyright 2011, IOP Publishing Ltd. (**c**) Experimental setup for the measurement of the interlayer photocurrent of the WSe_2_/graphene (W/G) heterostructure. (**d**) Transfer characteristics of the W/G photodiode. Reprinted with permission from [118]. Copyright 2018, American Chemical Society.

Semiconductor doping can modify semiconductors on the atom scale, which has been fully applied in optoelectronic device preparation with enhanced properties, such as NEMS sensors, photonics devices, sensors, and resonators. Liao et al. [117] modified 1D ZnO NWs field-effect transistors (FET) with a Ga^+^ treatment (Figure 13a). After 30 kV Ga^+^ irradiation, the switching ratio was improved by several orders of magnitude (Figure 13b) due to a decrease in the surface-trapped electrons and their concentration in the carrier. Nanda et al. [51] studied 2D BN encapsulated graphene (h-BN) and found that it exhibited n type conduction by controlling He^+^ irradiation. This was because the self-healing of the beam-induced lattice damage was promoted by unbound atoms between the sp^2^ layers of the graphene and h BN. Recently, Liu et al. [118] constructed vertical heterostructure WSe_2_/graphene (W/G) and found that its optical response could be enhanced by controlling the formation of point defects with Ga^+^ irradiation, as shown in Figure 13c,d. The fastest photo responsivity was about 0.6 ms, two orders of magnitude greater than that of pristine W/G. This provided an effective method for optimizing the performance of photoelectric devices based on vertical heterostructures. 

**Figure 14:**
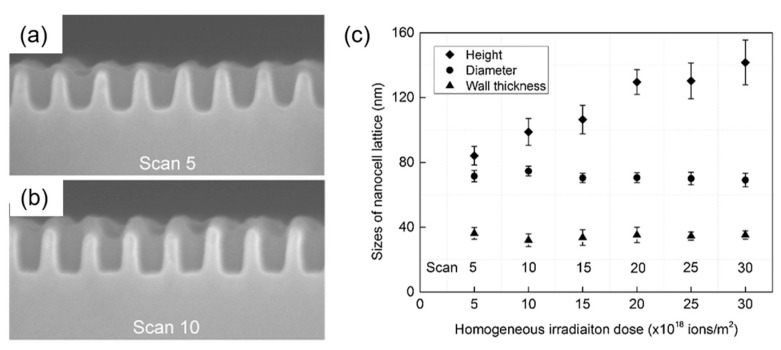
Cross-sectional SEM images of InSb nanocell lattices fabricated using 30 kV Ga^+^ FIB after (**a**) scan 5 and (**b**) scan 10; and (**c**) sizes of nanocell lattice versus beam scan. Reprinted with permission from [120]. Copyright 2016, Elsevier B.V.

FIB micromilling is also used to fabricate materials with complex 3D and high-aspect-ratio structures. Micro holes have shown potential in electrical devices, micro-fluidic devices, MEMS devices, chemical analyses in biochemistry, and so on [119]. A controllable 3D structure can be attained by adjusting the FIB-SEM parameters of acceleration voltage, dwell time, milling mode, pitch pixels, and sputtering yield, etc. Ishikawa et al. [120] fabricated a nanocell lattice with a high aspect ratio of two (cell height/cell diameter) on an InSb semiconductor surface (Figure 14a,b). They also found that the intermediate flux ion irradiation during a bottom-up process needs to be optimized for a high-aspect-ratio nanocell and it can be regulated by ion doses (Figure 14c).

### 3.3. Insulative Materials

Different from conductive and semiconductive materials, most insulative materials have a conductivity as low as 10^−7^ s/m. When a high-energy FIB bombards insulative materials, local charge accumulation will destroy the controllability of the FIB-SEM fabrication, especially on the sample surface, which makes SEM images fuzzy and distorted during the process of FIB writing [121]. In general, this local charge accumulation can be reduced by choosing a low current or coating an Au/Pt conductive layer on the samples [122]. However, it is impossible to guide all the local charges away, and the above methods may be of little use once the parameters of the incident beam are changed. Another way is to adjust the parameters of FIB and SEM to obtain the optimum secondary signals for fine SEM imaging [123]. In the following section, the 3D, high-precision, controllable fabrication of insulative materials mainly focuses on FIB milling in high-aspect-ratio structures, curved surfaces, and tomography.

**Figure 15:**
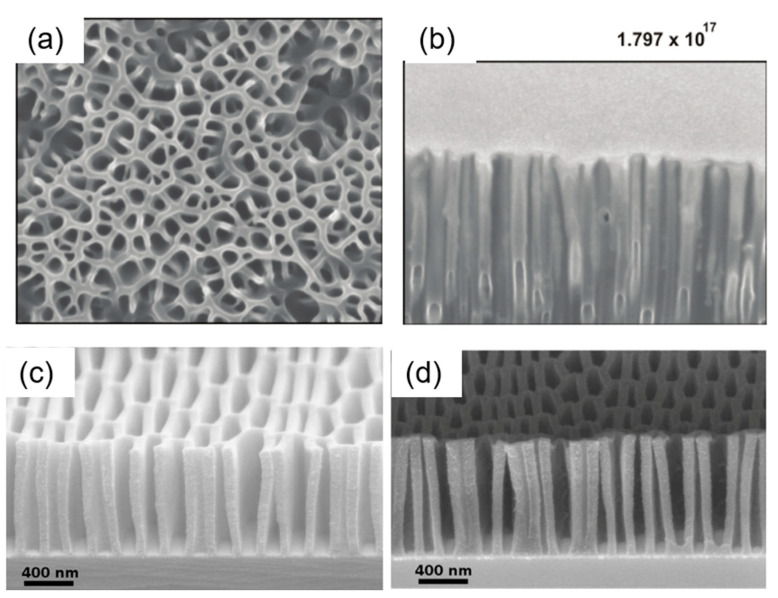
SEM images with different views of the nano-tunnel structures with a high aspect ratio (**a**) plan view; and (**b**) cross-section view. Reprinted with permission from [126]. Copyright 2009, IOP Publishing Ltd. SEM images of ~1 µm thick PMMA on Cr/Si substrate (**c**) Period = 200 nm, line bias = 15 nm; and (**d**) period = 160 nm, line bias = 25 nm. Reprinted with permission from [127]. Copyright 2009, Elsevier B.V.

Polymethyl methacrylate (PMMA) is taken as an example for studying a high-aspect-ratio structure milled by a FIB. PMMA has advantages of a high transparency, light weight, high mechanical strength, and easy processing, which makes it an ideal substrate for optical devices [124] and wearable electronic devices. High-precision machining based on PMMA with FIB-SEM can be achieved by adjusting the beam parameters such as the beam current, scanning strategy, and dwell time [125]. Her et al. [126] fabricated angled nano-scale tunnels with a high aspect ratio on PMMA (Figure 15), achieving a high aspect ratio of 700–1500 nm in the depth and 60 nm in the mean diameter with a 5 kV Ga^+^ ion beam and specific ion beam current. These structures show potential for creating a mold for anisotropic adhesives. Additionally, Gorelick et al. [127] fabricated nanostructures with high aspect ratios (>11) on thick PMMA resist (~1 µm) for applications in X-ray optical devices.

**Figure 16:**
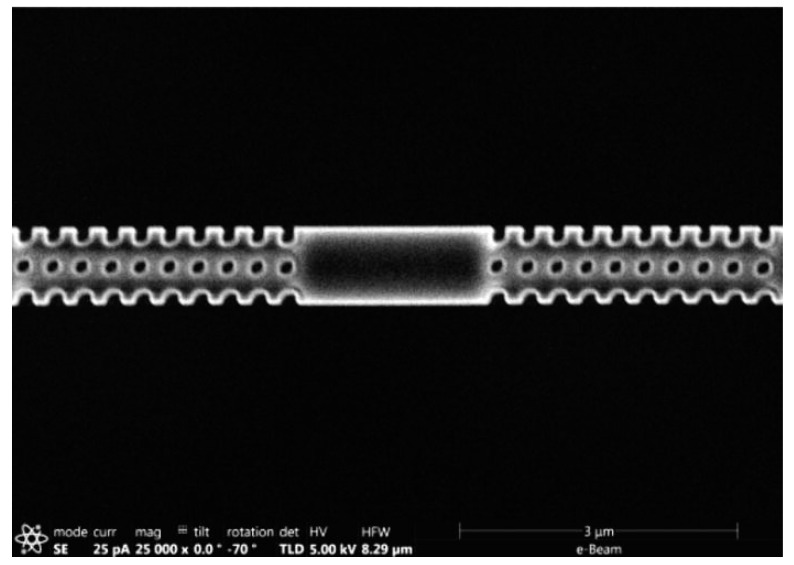
SEM image of a 970 nm diameter nanofiber-based milling using a Ga^+^ FIB. The measured size is ~141.6 nm × 130.4 nm with a pitch of ~326.3 nm. Reprinted with permission from [129]. Copyright 2017, AIP Publishing.

Great progress has been made in controllable preparation on the unconventional curved surfaces of optical fiber nanomaterials with a high precision, which extends FIB-SEM synchronization systems in various fiber-optic devices for near-field scanning optical microscopy (NSOM) fiber probes, plasmonic nano-arrays, and beam-shaping structures [36,128]. Li et al. [129] milled 970 nm diameter nanofiber-based optical cavities by controlling the scanning strategy precisely to obtain complex nanostructures (Figure 16). Kim et al. [130] obtained a fiber-optic localized surface plasmon resonances (LSPR) sensor using a combination of FIB milling and deposition technology. A gold film was deposited on the tip of a multimode optical fiber, then a nanodisk of a 66 × 66 array was prepared in a gold film patterned through FIB milling with an array gap of 200 nm.

The usage of the 3D controllable fabrication of FIB milling in tomography has been developed, which combines FIB layer-by-layer material removal at the atomic level and SEM imaging to reconstruct volumetric structures in 3D [62,131]. Generally, a lower beam current (0.15–1.5 nA) is used to depose a protective layer of Pt, C, or Au on the sample, then a higher beam current (15–45 nA) is chosen to mill the materials. Furthermore, FIB fabrication can be improved by polishing the sample surface with a low-energy and low-current beam to solve the uneven milling induced by the FIB beam tail [132]. At present, the 3D imaging of biomaterials at the nanoscale is a hotspot in nanobiology, and FIB-SEM synchronization technology contributes greatly to the 3D reconstruction of relatively large biomaterials with micrometer-scale thicknesses, such as entire cells or tissues. Heymann et al. [133] first reported the 3D volume reconstruction of biological specimens for yeast cells. Trebichalská et al. [134] reconstructed an unprecedented view of ooplasmic architecture and observed organelle distribution patterns in nine donor oocytes’ developmental competence. 3D image segmentation was performed to extract information. Xu et al. [135] expanded the image volume of FIB-SEM by more than four orders of magnitude, from 10^3^ µm^3^ to 3 × 10^7^ µm^3^, by choosing the enhanced FIB-SEM mode. Berger et al. [14] used an Ar plasma source to prepare slices with a success rate of 85% and provided a new way of obtaining a higher throughput 3D characterization of biomaterials with a pseudo-atomic resolution of 4.9 Å. FIB-SEM tomography also shows potential in the 3D reconstruction of porous materials with a controllable morphology and internal pore distributions. For example, Röding et al. [136] optimized the milling and imaging parameters for the soft porous polymer of ethyl cellulose films with a poor conductivity, and obtained an automatically segmented structure with a porosity as good as that of a manually segmented structure. Papynov et al. [72] reconstructed magnetic hematite structured porous ceramics using the SPS technique and obtained high-compactness ceramics with macropores of a 680–700 nm mean size.

## 4. Conclusions and Outlook

In this paper, we provided an overview of FIB technology, including its ion optical system, three operating modes, and FIB equipment combined with other systems in detail. The 3D, high-precision, controllable fabrication of conductive, semiconductive, and insulative materials was achieved with a FIB-SEM synchronization system by optimizing and modulating the FIB-SEM parameters and scanning modes. With the development of FIB-SEM synchronization technology, there still exists challenges in the 3D, controllable processing of flexible insulative materials using FIB-SEM with a high resolution. 

Except for the low conductivity presented in the paper, most flexible insulative materials have a low melting point, and a poor thermal conductivity usually with 0.01–0.1 W/(m·K), and hence severe damages such as shadowing effects on the cross-sectional surface, artefacts including curtaining and redeposition, local charging, low contrast, and sample heating will occur when a high-energy FIB bombards them. These factors need to be considered to attain the 3D, controllable processing of insulative materials with a higher resolution. In order to reduce the thermal damage, Bassim et al. [121] suggested coating Au/Pt as a radiator, but this method could only reduce the thermal effect to a certain extent and did not fully solve the rising temperature issue during the processing of polymers. Lee et al. [137] adopted an expensive, low-temperature sample stage to solve the thermal damage problem under a large beam current (~1000 pA), which protected an area of 200 nm from the ion beam bombardment at −25 °C, using a 30 keV 1000 pA Ga^+^ beam. Artefacts are inevitable due to ion beam implantation, which are dependent on the multiple parameters of ion species, ion energy, ion beam current, scanning strategy, dwell time, and sample composition, etc. Ar^+^ cleaning can only solve artefacts partly, by removing the degraded layer. In future, to realize the controllable, high-resolution, 3D fabrication of flexible insulative materials, a new dynamic model of dual-beam simultaneous fabrication with a rising temperature and local charge accumulation needs to be built for obtaining a relationship between the simultaneous beam, sample temperature, local charge accumulation field, and machining precision.

## Figures and Tables

**Table 1:** Commercial ion sources and related performance parameters.

Ion Source Type	Main Ion Species	$\;\;\;\beta_{r}\;\;\left(Am^{-2}sr^{-1}V^{-1}\right)$	$\Delta E_{\mathrm{FWHM}}(\mathrm{eV})$	Source Spot (nm)	Optional Ion Species
LMIS	Ga^+^	$1\times 10^{6}$	5	50–100	B, Be, Sn, Au …
GFIS	He^+^	$1\times 10^{9}$	1	1	Ne^+^
Plasma	Xe^+^ (ICP)	$1\times 10^{4}$	5	>400 nm	${\mathrm{Ar}}^{+}\;(\mathrm{ICP}),\;O_{2}^{+}$(ICP), He^+^ (ICP)

**Table 2:** 3D controllable FIB milling of semiconductive materials.

Materials	Structures	Sources	Applications	References
Porous-based graphene	Nanoporous	Ga^+^, He^+^	Membrane separation technology	[111]
h-BN	Nanograting	He^+^, Ne^+^	Nanomechanical switches driven by light	[112]
HSQ/Si	Nanograting	Ne^+^	Chemical sensing, magnetic storage	[113]
SnO_2_/In_2_O_3_	Nanowires	Ga^+^	Field-effect transistors (FET)	[114]
GaN	Micropillar	Ar^+^	FET	[115]
Si_3_N_4_	Nanostrings	Ga^+^	Nanogap electron	[116]

## Data Availability

This is a review paper not offering any original data.
